# Clustering gene expression time series data using an infinite Gaussian process mixture model

**DOI:** 10.1371/journal.pcbi.1005896

**Published:** 2018-01-16

**Authors:** Ian C. McDowell, Dinesh Manandhar, Christopher M. Vockley, Amy K. Schmid, Timothy E. Reddy, Barbara E. Engelhardt

**Affiliations:** 1 Computational Biology & Bioinformatics Graduate Program, Duke University, Durham, North Carolina, United States of America; 2 Center for Genomic & Computational Biology, Duke University, Durham, North Carolina, United States of America; 3 Department of Biostatistics & Bioinformatics, Duke University Medical Center, Durham, North Carolina, United States of America; 4 Biology Department, Duke University, Durham, North Carolina, United States of America; 5 Department of Computer Science, Princeton University, Princeton, New Jersey, United States of America; 6 Center for Statistics and Machine Learning, Princeton University, Princeton, New Jersey, United States of America; University of California Irvine, UNITED STATES

## Abstract

Transcriptome-wide time series expression profiling is used to characterize the cellular response to environmental perturbations. The first step to analyzing transcriptional response data is often to cluster genes with similar responses. Here, we present a nonparametric model-based method, Dirichlet process Gaussian process mixture model (DPGP), which jointly models data clusters with a Dirichlet process and temporal dependencies with Gaussian processes. We demonstrate the accuracy of DPGP in comparison to state-of-the-art approaches using hundreds of simulated data sets. To further test our method, we apply DPGP to published microarray data from a microbial model organism exposed to stress and to novel RNA-seq data from a human cell line exposed to the glucocorticoid dexamethasone. We validate our clusters by examining local transcription factor binding and histone modifications. Our results demonstrate that jointly modeling cluster number and temporal dependencies can reveal shared regulatory mechanisms. DPGP software is freely available online at https://github.com/PrincetonUniversity/DP_GP_cluster.

## Author summary

Transcriptome-wide measurement of gene expression dynamics can reveal regulatory mechanisms that control how cells respond to changes in the environment. Such measurements may identify hundreds to thousands of responsive genes. Clustering genes with similar dynamics reveals a smaller set of response types that can then be explored and analyzed for distinct functions. Two challenges in clustering time series gene expression data are selecting the number of clusters and modeling dependencies in gene expression levels between time points. We present a methodology, DPGP, in which a Dirichlet process clusters the trajectories of gene expression levels across time, where the trajectories are modeled using a Gaussian process. We demonstrate the performance of DPGP compared to state-of-the-art time series clustering methods across a variety of simulated data. We apply DPGP to published microbial expression data and find that it recapitulates known expression regulation with minimal user input. We then use DPGP to identify novel human gene expression responses to the widely-prescribed synthetic glucocorticoid hormone dexamethasone. We find distinct clusters of responsive transcripts that are validated by considering between-cluster differences in transcription factor binding and histone modifications. These results demonstrate that DPGP can be used for exploratory data analysis of gene expression time series to reveal novel insights into biomedically important gene regulatory processes.

This is a *PLOS Computational Biology* Methods Paper.

## Introduction

The analysis of time series gene expression has enabled insights into development [1–3], response to environmental stress [4], cell cycle progression [5, 6], pathogenic infection [7], cancer [8], circadian rhythm [9, 10], and other biomedically important processes. Gene expression is a tightly regulated spatiotemporal process. Genes with similar expression dynamics have been shown to share biological functions [11]. Clustering reduces the complexity of a transcriptional response by grouping genes into a small number of response types. Given a set of clusters, genes are often functionally annotated by assuming *guilt by association* [12], sharing sparse functional annotations among genes in the same cluster. Furthermore, regulatory mechanisms characterizing shared response types can be explored using these clusters by, for example, comparing sequence motifs or other features within and across clusters.

Clustering methods for time series transcription data partition genes into disjoint clusters based on the similarity of expression response. Many clustering methods, such as hierarchical clustering [11], k-means clustering [13], and self-organizing maps [14], evaluate response similarity using correlation or Euclidean distance. These methods assume that expression levels at adjacent time points are independent, which is invalid for transcriptomic time series data [15]. Some of these methods require a prespecified number of clusters, which may require model selection or post hoc analyses to determine the most appropriate number.

In model-based clustering, similarity is determined by how well the responses of any two genes fit the same generative model [15, 16]. Model-based methods thus define a cluster as a set of genes that is more likely to be generated from a particular cluster-specific model than other possible models [17]. Mclust, for example, assumes a Gaussian mixture model (GMM) to capture the mean and covariance of expression within a cluster. Mclust selects the optimal number of clusters using the Bayesian information criterion (BIC) [18]. However, Mclust does not take into account uncertainty in cluster number [19].

To address the problem of cluster number uncertainty, finite mixture models can be extended to infinite mixture models using a Dirichlet process (DP) prior. This Bayesian nonparametric approach is used in the Infinite Gaussian Mixture Model [20] and implemented in the tools Gaussian Infinite Mixture Models, or GIMM [21] and Chinese Restaurant Cluster, or CRC [22]. Using Markov chain Monte Carlo (MCMC) sampling, GIMM iteratively samples cluster-specific parameters and assigns genes to existing clusters, or creates a new cluster based on both the likelihood of the gene expression values with respect to the cluster-specific model and the size of each cluster [21]. An advantage of nonparametric models is that they allow cluster number and parameter estimation to occur simultaneously when computing the posterior. The DP prior has a “rich get richer” property—genes are assigned to clusters in proportion to the cluster size—so bigger clusters are proportionally more likely to grow relative to smaller clusters. This encourages varied cluster sizes as opposed to approaches that encourage equivalently sized clusters.

Clustering approaches for time series data that encode dependencies across time have also been proposed. SplineCluster models the time dependency of gene expression data by fitting non-linear spline basis functions to gene expression profiles, followed by agglomerative Bayesian hierarchical clustering [23]. The Bayesian Hierarchical Clustering (BHC) algorithm performs Bayesian agglomerative clustering as an approximation to a DP model, merging clusters until the posterior probability of the merged model no longer exceeds that of the unmerged model [24–26]. Each cluster in BHC is parameterized by a Gaussian process (GP). With this greedy approach, BHC does not capture uncertainty in the clustering.

Recently, models combining DPs and GPs have been developed for time series data analysis. For example, a recent method combines the two to cluster low-dimensional projections of gene expression [27]. The semiparametric Bayesian latent trajectory model was developed to perform association testing for time series responses, integrating over cluster uncertainty [28]. Other methods using DPs or approximate DPs to cluster GPs for gene expression data use different parameter inference methods [25, 27, 29]. However, several methods similar to DPGP lack software to enable application of the methods by biologists or bioinformaticians [27, 29].

Here we develop a statistical model for clustering time series data, the Dirichlet process Gaussian process mixture model (DPGP), and we package this model in user-friendly software. Specifically, we combine DPs for incorporating cluster number uncertainty and GPs for modeling time series dependencies. In DPGP, we explore the number of clusters and model the time dependency across gene expression data by assuming that gene expression for genes within a cluster are generated from a GP with a cluster-specific mean function and covariance kernel. A single clustering can be selected according to one of a number of optimality criteria. Additionally, a matrix is generated that contains estimates of the posterior probability that each pair of genes belongs to the same cluster. Missing data are naturally incorporated into this GP framework, as are observations at unevenly spaced time points. If all genes are sampled at the same time points with no missing data, we leverage this fact to speed up the GP regression task in a fast version of our algorithm (fDPGP).

To demonstrate the applicability of DPGP to gene expression response data, we applied our algorithm to simulated, published, and original transcriptomic time series data. We first applied DPGP to hundreds of diverse simulated data sets, which showed favorable comparisons to other state-of-the-art methods for clustering time series data. DPGP was then applied to a previously published microarray time series data set, recapitulating known gene regulatory relationships [30]. To enable biological discovery, RNA-seq data were generated from the human lung epithelial adenocarcinoma cell line A549 from six time points after treatment with dexamethasone (dex) for up to 11 hours. By integrating our DPGP clustering results on these data with a compendium of ChIP-seq data sets from the ENCODE project, we reveal novel mechanistic insights into the genomic response to dex.

## Results

### DPGP compares favorably to state-of-the-art methods on simulated data

We tested whether DPGP recovers true cluster structure from simulated time series data. We applied DPGP and the fast version of DPGP, fDPGP, to 620 data sets generated using a diverse range of cluster sizes and expression traits (S1 Table). We compared our results against those from BHC [25], GIMM [21], hierarchical clustering by average linkage [11], k-means clustering [13], Mclust [18], and SplineCluster [23]. To compare observed partitions to true partitions, we used *Adjusted Rand Index* (ARI), which measures the similarity between a test clustering and ground truth in terms of cluster agreement for element pairs [31, 32]. ARI is 1 when two partitions agree exactly and 0 when two partitions agree no more than is expected by chance [31, 32]. ARI was recommended in a comparison of metrics [33] and has been used to compare clustering methods in similar contexts [21, 34–36].

**Fig 1:**
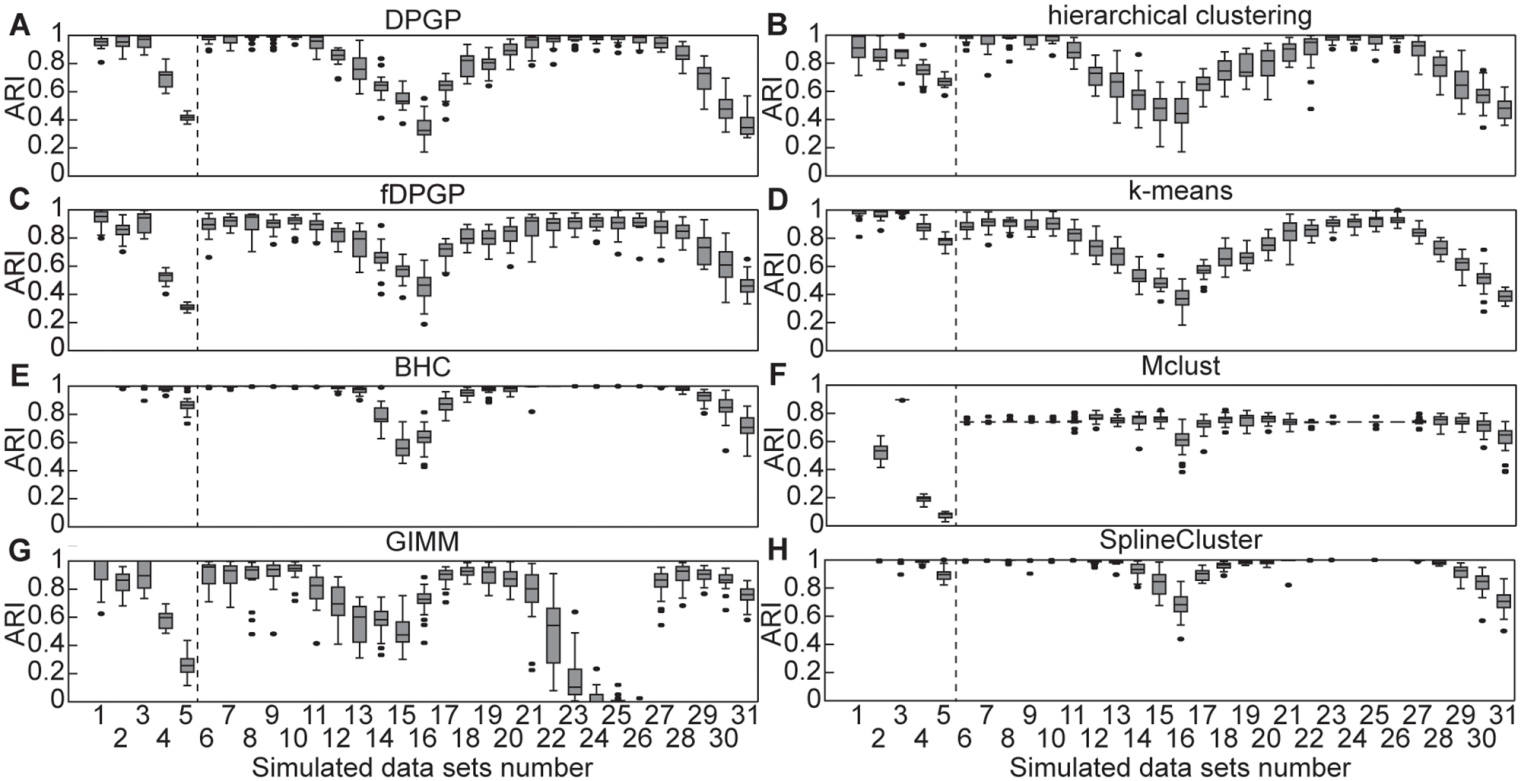
Clustering performance of state-of-the-art algorithms on simulated time series data. Box plots show summaries of the empirical distribution of clustering performance for (A) DPGP, (B) hierarchical clustering, (C) fDPGP, (D), k-means clustering, (E) BHC, (F) Mclust, (G) GIMM, and (H) SplineCluster in terms of Adjusted Rand Index (ARI) across twenty instances of each of the 31 data set types (S1 Table). Higher values represent better recovery of the simulated clusters. Vertical dotted lines separate data sets with widely varied cluster size distributions (*left*) from data sets with widely varied generating hyperparameters (*right*). Observations that lie beyond the first or third quartile by 1.5× the interquartile range are shown as outliers.

Assuming GPs as generating distributions, we simulated data sets with varied cluster size distributions, length scale, signal variance, and marginal variance (S1 Table). Across simulations, DPGP generally outperformed GIMM, k-means, and Mclust, but was generally outperformed by BHC and SplineCluster, and performed about as well as hierarchical clustering (Fig 1 and S2 Table). fDPGP performed nearly as well as DPGP (Fig 1C). The performance of hierarchical clustering and k-means benefited from prespecification of the true number of clusters—with a median number of 24 clusters across simulations—while the other methods were expected to discover the true number of clusters with no prior specification. In scientific applications, *a priori* knowledge of the optimal number of clusters is unavailable, necessitating multiple runs and post hoc analyses for hierarchical clustering and k-means. Methods that do not model temporal dependencies in observations—GIMM, k-means, and Mclust—performed worst in our evaluations, suggesting that there is substantial value in explicitly modeling temporal dependencies.

We simulated an additional 500 data sets with *t*-distributed errors (*df* = 2), which is a heavier-tailed distribution than the Gaussian and may more realistically reflect the distribution of quantified gene expression levels in RNA-seq data [37]. Again, we varied cluster size, length scale, and signal variance (S1 Table). In these simulations, DPGP outperformed BHC, SplineCluster, Mclust, and hierarchical clustering, and was outperformed by GIMM and k-means clustering (S1 Fig and S2 Table). DPGP and fDPGP performed nearly the same (Wilcoxon two-sided signed-rank, *p* = 0.12). Across all simulations, performance depended on the assumptions of the simulated data and no algorithm outperformed all others across all data sets. DPGP performed well under both Gaussian- and *t*-distributed error models, which demonstrates robustness to model assumptions.

DPGP successfully recovered true cluster structure across a variety of generating assumptions except in cases of a large number of clusters each with a small number of genes (data sets 4 and 5) or a small signal variance (data set 16) and a high marginal variance (data set 31; Fig 1). It is possible that DPGP performed poorly on data sets with many clusters, each with a small number of genes, because this kind of cluster size distribution poorly matches the DP prior. The DP prior may not be appropriate for all clustering applications. However, BHC, which also assumes a DP prior, performed quite well on these data sets. Moreover, clustering a large number of genes (500) into a large number of clusters (100) might best be performed using other types of methods [38]

For each gene, DPGP can optionally estimate a probability of inclusion to its assigned cluster based on the weighted mean frequency of co-occurrence with all other genes in that cluster across Gibbs samples. Performance of DPGP on the data sets with Gaussian-distributed error improved after omitting genes with low probability cluster assignments both across all data sets and across the ten data set classes for which DPGP performed worst (Wilcoxon two-sided signed-rank, including only genes with probability of inclusion of, e.g., ≥ 0.7 versus all genes, *p* ≤ 2.2 × 10^−16^; S2A and S2C Fig). Performance did not improve when cluster assignment probabilities were permuted across genes (*p* > 0.21, S2B and S2D Fig). After excluding genes with cluster inclusion probabilities < 0.9, there was an improvement in performance for data sets with small signal variance (data sets 16 and 17) or high marginal variance (data sets 30 and 31), but minimal improvement on data sets with a large number of clusters, each with a small number of genes (data sets 4 and 5; S2E Fig). These results imply that DPGP generates useful cluster assignment probabilities.

The algorithms tested varied greatly in speed. On moderately sized data sets (≲ 1,000 genes), fDPGP was substantially faster than GIMM and BHC, but slower than hierarchical clustering, k-means, Mclust, and SplineCluster [S3A Fig; Wilcoxon two-sided signed-rank (WSR), DPGP versus each method, *p* ≤ 8.86 × 10^−5^]. On larger data sets of up to 10,000 genes, fDPGP again was faster than BHC and GIMM (S3B Fig; WSR, DPGP versus each method, *p* ≤ 5.06 × 10^−3^). BHC failed to cluster data sets with ≥ 2,000 genes within 72 hours. Because of the speed and reliable clustering performance of fDPGP, we use this version in the biological data applications below.

An important advantage of DPGP, as a probabilistic method, is that uncertainty in clustering and cluster trajectories is captured explicitly. Some implications of the probabilistic approach are that cluster means and variances can be used to quantify the likelihood of future data, to impute missing data points at arbitrary times, and to integrate over uncertainty in the cluster assignments [39]. Using these same data simulations, we clustered expression trajectories while holding out each of the four middle time points of eight total time points. We computed the proportion of held-out test points that fell within the 95% credible intervals (CIs) of the estimated cluster means. For comparison, we also permuted cluster membership across all genes 1,000 times and recomputed the same proportions. We found that DPGP provided accurate CIs on the simulated gene expression levels (S4 Fig). Across all simulations, at least 90% of test points fell within the estimated 95% CI, except for data set types with large length-scales or high signal variances (both parameters ∈ {1.5, 2, 2.5, 3}). The proportion of test points that fell within the 95% CIs was consistently higher for true clusters than for permuted clusters [Mann-Whitney U-test (MWU), *p* ≤ 2.24 × 10^−6^], except for data with small length scales ({0.1, …, 0.5}) when the proportions were equivalent (MWU, *p* = 0.24). This implies that the simulated sampling rates in these cases were too low for DPGP to capture temporal patterns in the data.

For the simulations with Gaussian-distributed error, in which DPGP performed worse than BHC or SplineCluster with respect to recovering the true cluster structure, the clusters inferred from the data provided useful and accurate CIs for unseen data. For example, DPGP performed decreasingly well as the marginal variance was increased to 0.4, 0.5, and 0.6. However, the median proportions of test points within the 95% CIs were 93.4%, 92.6%, 91.9%, respectively (S4 Fig). This suggests that DPGP provides well calibrated CIs on expression levels over the time course and can theoretically be used for reliable imputation at arbitrary time points.

DPGP may also be used to evaluate the confidence in a specific clustering with respect to the fitted model, which can be important for revealing instances when many different partitions model the data nearly as well as one another. For example, across our simulated datasets, when DPGP did not precisely recover the cluster structure, we found there was also substantial uncertainty in the optimal partition. Specifically, the posterior probability of the oracle clustering with respect to the simulated observations was greater than both the posterior probability of the DPGP MAP partition and than the mean posterior probability across all DPGP samples in only 1.6% of cases (Z-test, *p* < 0.05). This suggests that, in nearly all simulated examples, the posterior probability was not strongly peaked at the true partition.

### Clustering oxidative stress transcriptional responses in a microbial model organism recapitulates known biology

Given the performance of DPGP on simulated data with minimal user input and no prespecification of cluster number, we next sought to assess the performance of DPGP on biological data. As a test case, we applied DPGP to published data from a single-celled model organism with a small genome (*Halobacterium salinarum*; 2.5 Mbp and 2,400 genes) exposed to oxidative stress induced by addition of H_2_O_2_ [30]. This multifactorial experiment tested the effect of deletion of the gene encoding the transcription factor (TF) RosR, which is a global regulator that enables resilience of *H. salinarum* to oxidative stress [40]. Specifically, transcriptome profiles of a strain deleted of the *rosR* gene (Δ*rosR*) and control strain were captured with microarrays at 10–20 minute intervals following exposure to H_2_O_2_. In the original study, 616 genes were found to be differentially expressed (DEGs) in response to H_2_O_2_, 294 of which were also DEGs in response to *rosR* mutation. In previous work, the authors clustered those 294 DEGs using k-means clustering with *k* = 8 (minimum genes per cluster = 13; maximum = 86; mean = 49) [30].

**Fig 2:**
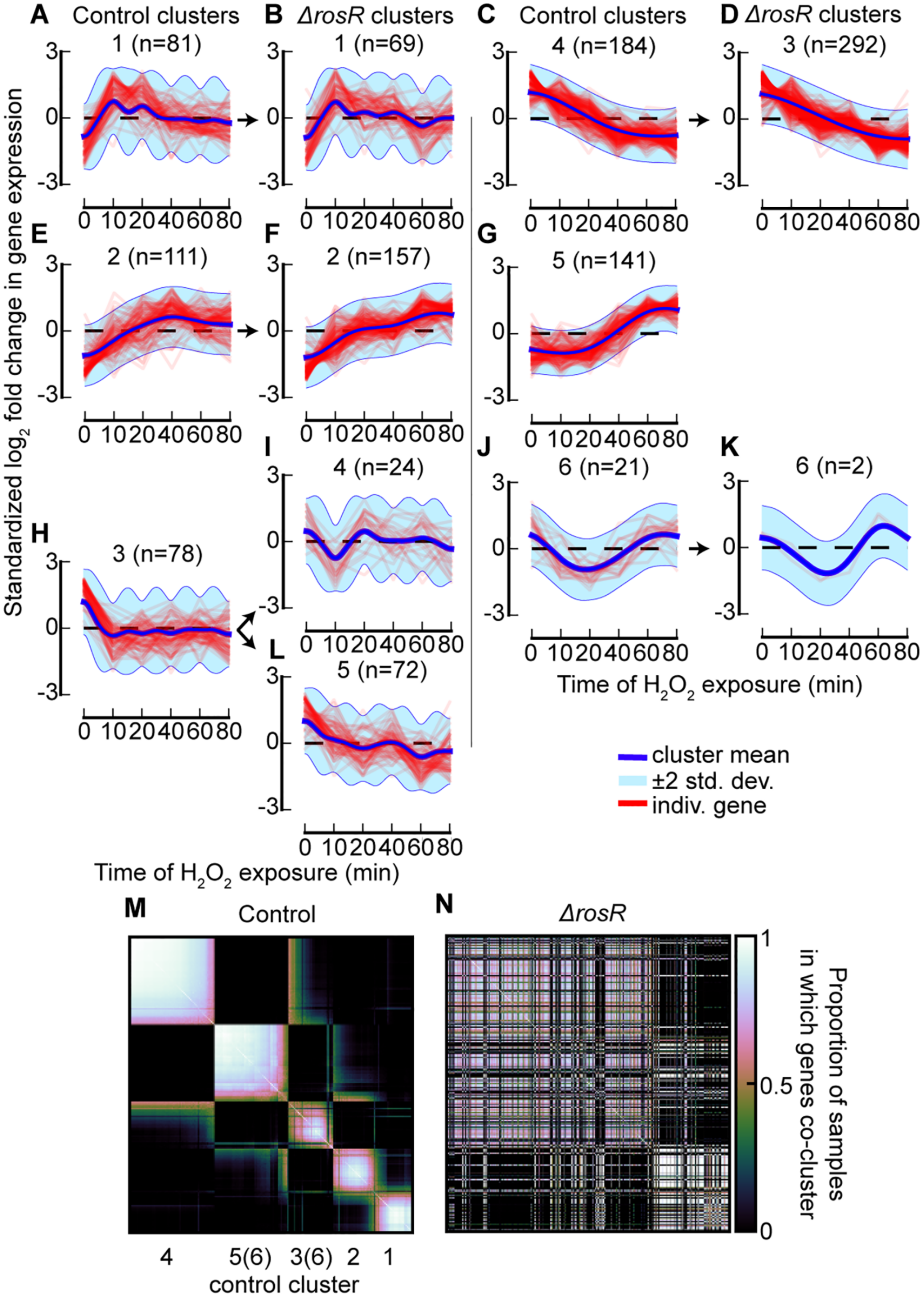
DPGP clusters in *H. salinarum* H_2_O_2_-exposed gene expression trajectories. (A–L) For each cluster, standardized log_2_ fold change in expression from pre-exposure levels is shown for each gene as well as the posterior cluster mean ±2 standard deviations. Control strain clusters are on left and Δ*rosR* clusters on right, organized to relate the Δ*rosR* clusters that correspond to each control cluster. Note that control cluster 5 had no corresponding Δ*rosR* cluster, but transcripts in this cluster instead distribute to a variety of Δ*rosR* clusters, none of which had a majority of cluster 5 transcripts. (M) Heatmap displays the proportion of DPGP samples from the Markov chain in which each gene (on the rows and columns) clusters with every other gene in the control strain. Rows and columns were clustered by Ward’s linkage. The predominant blocks of elevated co-clustering are labeled with the control cluster numbers to which the genes that compose the majority of the block belong. As indicated, cluster 6 is dispersed across multiple blocks, primarily the blocks for clusters 3 and 5. (N) Same as (M), except that values are replaced by the proportions in the Δ*rosR* strain instead of the control strain. Rows and columns ordered as in (M).

We used DPGP on these *H. salinarum* time series data to cluster expression trajectories from the 616 DEGs in each strain independently, which resulted in six clusters per strain when we consider the maximum *a posteriori* (MAP) partition (Fig 2). The number of genes in clusters from DPGP varied widely across clusters and strains (minimum genes per cluster = 2; maximum = 292; mean = 102.7) with greater variance in cluster size in trajectories from the mutant strain. To assess how DPGP clustering results compared to previous results using k-means, we focused on how the deletion of *rosR* affected gene expression dynamics. Out of the 616 DEGs, 372 moved from a cluster in the control strain to a cluster with a different dynamic trajectory in Δ*rosR* (e.g., from an up-regulated cluster under H_2_O_2_ in control, such as cluster 5, to a down-regulated cluster in Δ*rosR*, such as cluster 3; Fig 2 and S3 Table). Of these 372 genes, 232 were also detected as differentially expressed in our previous study [30] [significance of overlap, Fisher’s exact test (FET), *p* ≤ 2.2 × 10^−16^]. Comparing these DPGP results to previous analyses, similar fractions of genes were found to be directly bound by RosR according to ChIP-chip data from cells exposed to H_2_O_2_ for 0, 10, 20, and 60 minutes [40]. When all RosR binding at all four ChIP-chip time points were considered together, 8.9% of DPGP genes changing clusters were bound, similar to the 9.5% of DEGs that were bound in the previous analysis [30].

Genes most dramatically affected by deletion of *rosR* were those up-regulated after 40 minutes of H_2_O_2_ exposure in the control strain. For example, all 141 genes in control cluster 5 changed cluster membership in the Δ*rosR* strain (Fig 2; FET, *p* ≤ 2.2 × 10^−16^). Of these 141 genes up-regulated in the control strain in response to H_2_O_2_, 89 genes (63%) exhibited inverted dynamics, changing to down-regulated in the Δ*rosR* strain. These 89 genes grouped into two clusters in the Δ*rosR* strain (Δ*rosR* clusters 3 and 5; Fig 2 and S3 Table). The transcriptional effect of *rosR* deletion noted here accurately reflects previous observations: 84 of these 89 genes showed differential trajectories in the control versus Δ*rosR* strains previously [30]. RosR is required to activate these genes in response to H_2_O_2_ [30]. These results suggest that DPGP analysis accurately recapitulates previous knowledge of RosR-mediated gene regulation in response to H_2_O_2_ with reduced user input.

### DPGP reveals mechanisms underlying the glucocorticoid transcriptional response in a human cell line

Given the performance of DPGP in recapitulating known results for biological data, we next used DPGP for analysis of novel time series transcriptomic data. Specifically, we used DPGP to identify co-regulated sets of genes and candidate regulatory mechanisms in the human glucocorticoid (GC) response. GCs, such as dex, are among the most commonly prescribed drugs for their anti-inflammatory and immunosuppressive effects [41]. GCs function in the cell primarily by affecting gene expression levels. Briefly, GCs diffuse freely into cells, where they bind to and activate the glucocorticoid receptor (GR). Once bound to its ligand, GR translocates into the nucleus, where it binds DNA and regulates expression of target genes. The induction of expression from GC exposure has been linked to GR binding [42, 43]. However, while there are a plethora of hypotheses regarding repression and a handful of well-studied cases [44, 45], it has proved difficult to associate repression of gene expression levels with genomic binding on a genome-wide scale [42, 43]. Further, GC-mediated expression responses are far more diverse than simple induction or repression, motivating a time course study of these complex responses [46–50].

To characterize the genome-wide diversity of the transcriptional response to GCs and to reveal candidate mechanisms underlying those responses, we performed RNA-seq in the human lung adenocarcinoma-derived A549 cell line after treatment with the synthetic glucocorticoid (GC) dex at 1, 3, 5, 7, 9, and 11 hours, resulting in six time points. This data set is among the most densely sampled time series of the dex-mediated transcriptional response in a human cell line.

#### DPGP clustered transcriptional responses into four predominant clusters

**Fig 3:**
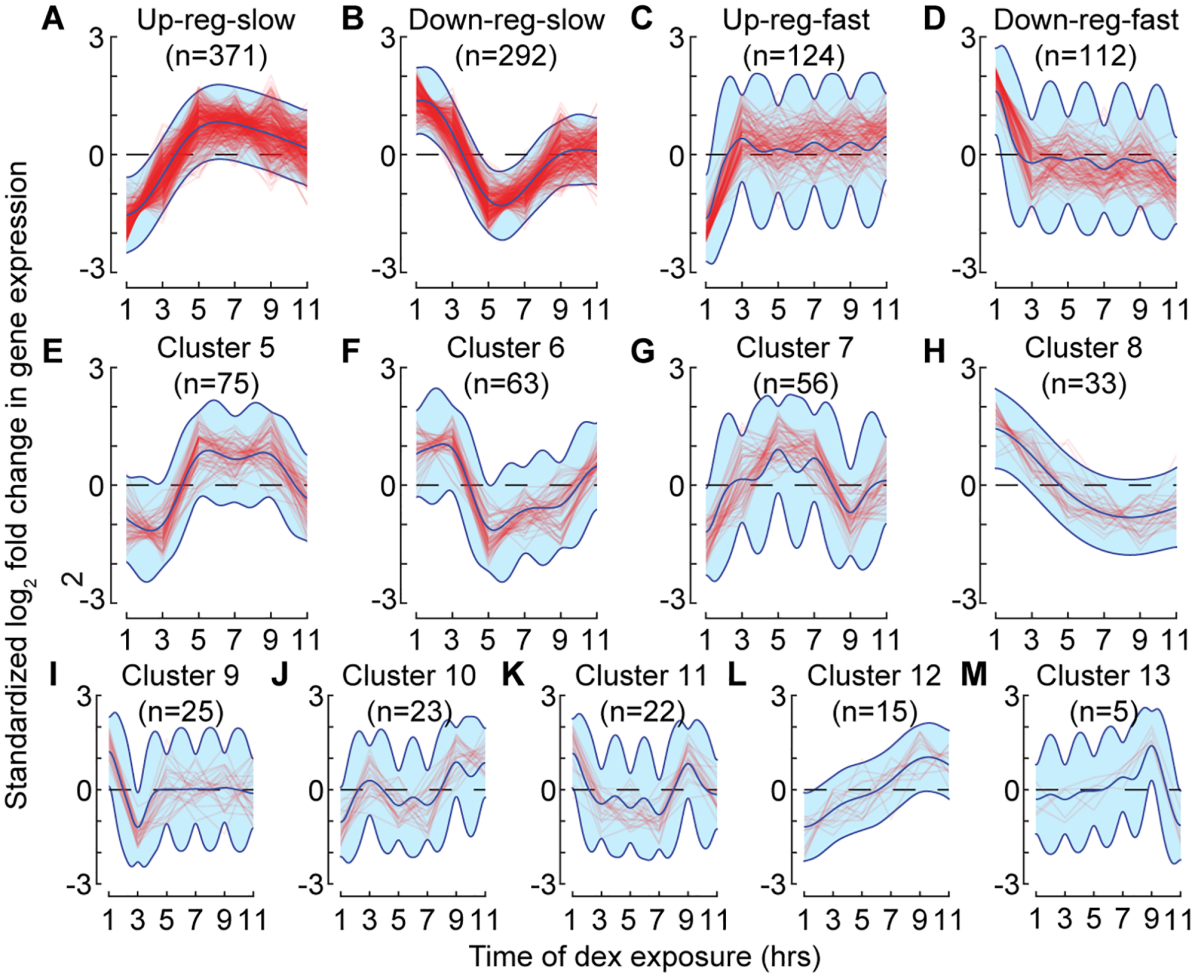
Clustered trajectories of differentially expressed transcripts in A549 cells in response to dex. For each cluster in (A–M), standardized log_2_ fold change in expression from pre-dex exposure levels is shown for each transcript, and the posterior cluster mean and ±2 standard deviations according to the cluster-specific GP.

We used DPGP to cluster 1,216 transcripts that were differentially expressed at two consecutive time points (FDR ≤ 0.1). DPGP found 13 clusters with a mean size of 119 transcripts and a standard deviation of 108 transcripts (Fig 3 and S5 Fig). In order to analyze the shared mechanisms underlying expression dynamics for genes within a cluster and to validate cluster membership, we chose to study genes in the four largest clusters using a series of complementary analyses and data. These four clusters included 74% of the dex-responsive transcripts. We designated these clusters *up-reg-slow*, *down-reg-slow*, *up-reg-fast*, and *down-reg-fast* (Fig 3) where *fast* clusters had a maximal difference in mean expression levels between 1 and 3 hours and *slow* clusters had a maximal difference between 3 and 5 hours. A variety of other clusters were identified with diverse dynamics, revealing the complexity of the GC transcriptional response (Fig 3).

#### DPGP dex-responsive expression clusters differ in biological processes

Genes involved in similar biological processes often respond similarly to stimuli [11]. To determine if the DPGP clusters were enriched for genes that are jointly involved in biological processes, we tested each cluster for enrichment of Gene Ontology slim (GO-slim) biological process terms [51]. The *down-reg-slow* cluster was enriched for cell cycle-related terms such as *cell cycle*, *cellular aromatic compound metabolic process*, *heterocycle metabolic process*, *chromosome segregation*, and *cell division*, among other associated terms (see S4 Table for p-values). This cluster included genes critical to cell cycle progression such as *BRCA2*, *CDK1*, *CDK2*, and others. The down-regulation of these genes is consistent with the antiproliferative effects of GCs [52–54]. In contrast, the *down-reg-fast* cluster was enriched for terms related to *developmental process* such as *anatomical structure formation involved in morphogenesis* (S4 Table). Genes in the *down-reg-fast* cluster that were annotated as *anatomical structure formation involved in morphogenesis* included homeobox genes like *EREG*, *HNF1B*, *HOXA3*, and *LHX1* as well as growth factors like *TGFA* and *TGFB2*. Our results suggest that GC exposure in A549 cells leads to a rapid down-regulation of growth-related TFs and cytokines, and a slower down-regulation of crucial cell cycle regulators.

The *up-reg-slow* and *up-reg-fast* clusters did not differ substantially in functional enrichment, and both were enriched for *signal transduction*. Up-regulated genes annotated as *signal transduction* included multiple MAP kinases, *JAK1*, *STAT3* and others. Whereas the *down-reg-slow* cluster was enriched for genes annotated as *heterocycle metabolic process*, the *up-reg-slow* cluster was depleted (S4 Table). Overall, clustering enabled improved insight into GC-mediated transcriptional responses. Our results suggest that a novel functional distinction may exist between rapidly and slowly down-regulated genes.

#### DPGP clusters differ in TF and histone modification occupancy prior to dex exposure

We validated the four major expression clusters by identifying distinct patterns of epigenomic features that may underlie differences in transcriptional response to GC exposure. In particular, we looked to see whether the co-clustered genes had similar TF binding and chromatin marks before dex exposure. We hypothesized that similar transcriptional responses were driven by similar regimes of TF binding and chromatin marks. To test this, we used all ChIP-seq data generated by the ENCODE project [55] that were assayed in the same cell line and treatment conditions (S5 Table). For each data set and each transcript, we counted pre-aligned ChIP-seq reads in three bins of varied distances from the transcription start site (TSS; < 1 kb, 1–5 kb, 5–20 kb), based on evidence that suggests that different TFs and histone modifications function at different distances from target genes [56]. Both TF binding and histone modification occupancy are well correlated [57, 58]. In order to predict cluster membership of each transcript based on a parsimonious set of TFs and histone modifications in control conditions, we used elastic net logistic regression, which tends to include or exclude groups of strongly correlated predictors using a sparse logistic model [59]. We controlled for differences in basal expression prior to dex exposure by including the baseline transcription level as a covariate in the model.

**Fig 4:**
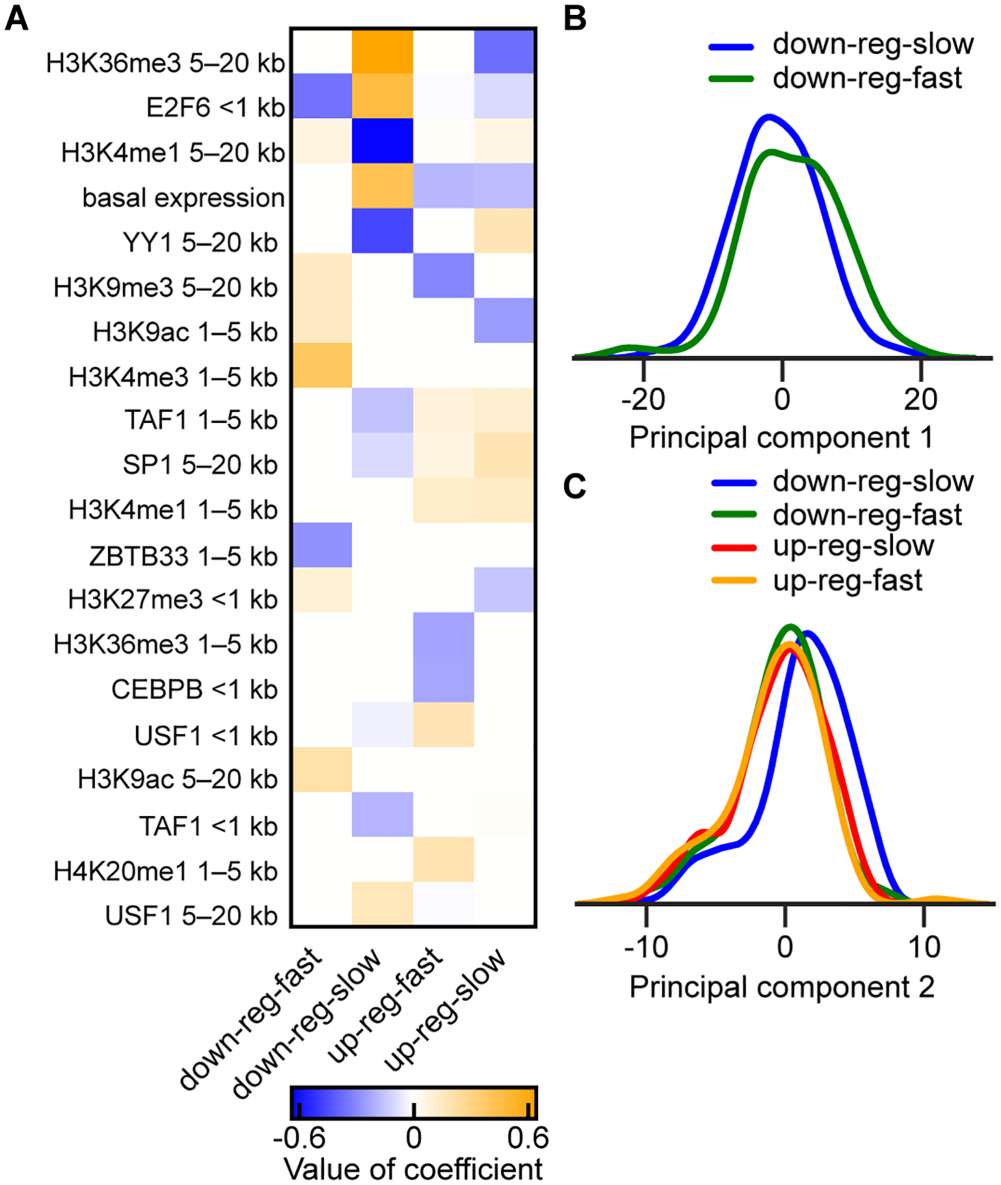
Differences in TF binding and histone modification occupancy in A549 cells in control conditions for the four largest DPGP clusters. (A) Heatmap shows the elastic net logistic regression coefficients for the top twenty predictors (sorted by sum of absolute value across clusters) of cluster membership for the four largest clusters. Predictors were log_10_ library size-normalized binned counts of ChIP-seq TF binding and histone modification occupancy in control conditions. Distance indicated in row names represents the bin of the predictor (e.g., <1 kb means within 1 kb of the TSS). An additional 23 predictors with smaller but non-zero coefficients are shown in S6 Fig. (B) Kernel density histogram smoothed with a Gaussian kernel and Scott’s bandwidth [63] of the TF binding and histone modification occupancy log_10_ library size-normalized binned count matrix in control conditions transformed by the first principal component (PC1) for the two largest down-regulated DPGP clusters. (C) Same as (B), but with matrix transformed by PC2 and with the four largest DPGP clusters.

The features that were most predictive of cluster membership—indicating an association with expression dynamics—were distal H3K36me3, promoter-proximal E2F6, and distal H3K4me1 (Fig 4A and S6 Fig). H3K36me3 marks the activity of transcription, and is deposited across gene bodies, particularly at exons [60, 61]. Its strength as a predictor of cluster membership may represent differences in the methylation of H3K36 between clusters of genes or, alternatively, residual differences in basal expression. E2F6 functions during G1/S cell cycle transition [62] and its binding was greater in the *down-reg-slow* cluster, which is consistent with the enrichment of genes with cell cycle biological process terms in the same cluster. H3K4me1 correlates strongly with enhancer activity [57] and the negative coefficient in our model for the *down-reg-slow* cluster suggests that the contribution of enhancers to expression differs across clusters (Fig 4).

The two large down-regulated clusters differed substantially in TF binding and histone modifications before exposure to dex (Fig 4A). To confirm, we ran the same regression model after limiting prediction to transcripts in those two clusters. We found that distal H3K4me1 and promoter-proximal E2F6 were highly predictive features, and also four distal histone features that have all been associated with enhancer activity (S7 Fig) [64, 65]. This analysis suggests predictive mechanistic distinctions between quickly and slowly down-regulated transcriptional responses to GC exposure. We performed elastic net regression to identify differential epigenomic features across only the two large up-regulated transcript clusters. No TFs or histone marks were differentially enriched in the up-regulated clusters, meaning that no covariates improved log loss by more than one standard error. Differences in regulatory mechanisms between the two clusters may involve downstream events not reflected in the ChIP data used here.

One drawback of our approach for studying clusters using enriched epigenomic features is that observations are available for only a handful of such epigenomic features for a specific cell type, and these covariates are often highly correlated [57, 58]. In the context of elastic net, results should be stable upon repeated inclusion of identical predictors in replicated models [59]. However, the variables identified as predictive may derive their predictiveness from their similarity to underlying causative TFs or histone modifications. To address the problem of correlated predictors, we used a complementary approach to reveal functional mechanisms distinguishing the four major expression clusters. We projected the correlated features of the standardized control TF and histone modification occupancy data onto a set of linearly uncorrelated covariates using principal components analysis (PCA). We then compared the clusters after transforming each gene’s epigenomic mappings by the two principal axes of variation, which were selected according to the scree plot method [66] (S8 Fig).

The first principal component (PC1) explained 47.9% of the variance in the control ChIP-seq data (S8 Fig). The 42 ChIP-seq covariates with the highest magnitude loadings on PC1 were restricted to distal, non-promoter TF binding and activation-associated histone mark occupancy, implicating enhancer involvement (for the value of all loadings on PC1, see S6 Table). Specifically, the features with the two highest magnitude loadings on PC1 were both binned counts of distal p300 binding, a histone acetyltransferase that acetylates H3K27 and is well established as an enhancer mark [57, 67].

We next compared the four largest clusters with respect to their projections onto PC1. We found that the *down-reg-slow* cluster differed substantially from the *down-reg-fast* cluster when transformed by PC1 (MWU, *p* ≤ 2.28 × 10^−3^; Fig 4B), while no other pairwise comparison was significant (MWU, *p* > 0.13). These results suggest that, in aggregate, slowly responding down-regulated transcripts have reduced enhancer activity in control conditions relative to quickly responding down-regulated transcripts.

The second principal component (PC2) explained 11.1% of the variance in the control ChIP-seq data (S8 Fig). The 21 ChIP-seq features with the greatest contributions to PC2 captured TF binding and activation-associated histone modifications within the promoter (S6 Table). By comparing the four largest clusters, we found that the *down-reg-slow* cluster differed from all other clusters with respect to PC2 (MWU, *p* ≤ 9.15 × 10^−7^; Fig 4C), while no other pairwise difference was significant (MWU, *p* > 0.28). These results illustrate that the slowly responding down-regulated transcripts collectively showed increased pre-dex promoter activity compared to the other three largest clusters.

#### Transcriptional response clusters show differences in dynamic TF and histone modification occupancy

**Fig 5:**
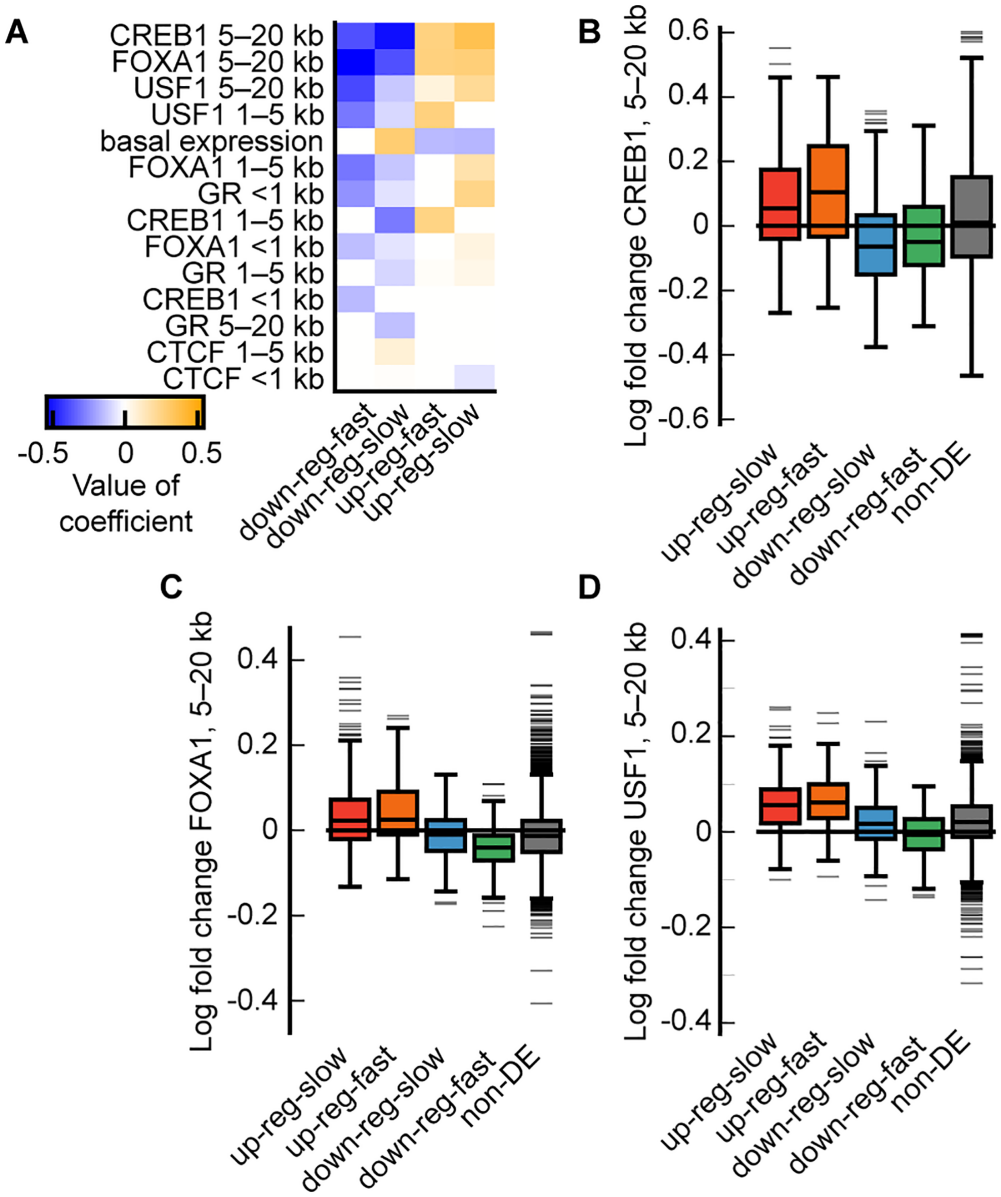
Differences in changes in transcription factor binding in A549 cells in response to glucocorticoid exposure for the four largest DPGP clusters. (A) Heatmap shows all coefficients (sorted by sum of absolute value across clusters) for predictors with non-zero coefficients as estimated by elastic net logistic regression of cluster membership for the four largest DPGP clusters. Predictors on y-axis represent log fold-change in normalized binned counts of TF binding from ethanol to dex conditions as assayed by ChIP-seq. Distance indicated in row names reflects the bin of the predictor (e.g., 1 kb = within 1 kb of TSS). (B) Boxplots show the logFC in normalized binned counts across clusters and for the group of non-DE transcripts for CREB1, (C) FOXA1, and (D) USF1.

We next validated our four largest dynamic expression clusters by examining the within-cluster similarity in changes in TF binding over time. To do this, we computed the log fold change in normalized ChIP-seq counts for all TFs (CREB1, CTCF, FOXA1, GR, and USF1) assayed through ENCODE with and without 1 hour treatment with 100 nM dex (S5 Table) [55]. We again fit an elastic net logistic regression model, this time to identify the changes in TF binding that were predictive of cluster. The most predictive features of cluster membership were changes in CREB1, FOXA1, and USF1 binding 5–20 kb from the TSS (Fig 5A). CREB1, FOXA1, and USF1 are all known transcriptional activators [68–70].

Increased binding of transcriptional activators was associated with increased expression and with more rapidly increased expression, while decreased binding was associated with decreased expression and more rapidly decreased expression. Specifically, genes in both up-regulated clusters had higher median log fold change in binding of CREB1, FOXA1, and USF1 compared to the two down-regulated clusters (MWU, *p* ≤ 1.5 × 10^−9^, Fig 5B–5D). Down-regulated clusters had lower median log fold change in the binding of certain TFs than the group of non-DE transcripts (CREB1 *down-reg-slow* versus non-DE, MWU, *p* ≤ 2.07 × 10^−15^, Fig 5C; CREB1, FOXA1, and USF1 *down-reg-fast* versus non-DE, MWU, *p* = 3.18 × 10^−5^, Fig 5B–5D). Additionally, the *down-reg-fast* cluster had lower median log fold change than the *down-reg-slow* cluster in FOXA1 and USF1 binding (MWU, *p* = 8.24 × 10^−6^, *p* = 1.29 × 10^−4^, respectively). Our results suggest that differences in TF binding over time may underlie differences in dynamic transcriptional response both in terms of up-regulation versus down-regulation and also in the speed of the transcriptional response.

## Discussion

We developed a Dirichlet process Gaussian process mixture model (DPGP) to cluster measurements of genomic features such as gene expression levels over time. We showed that our method effectively identified disjoint clusters of time series gene expression observations using extensive simulations. DPGP compares favorably to existing methods for clustering time series data, is robust to non-Gaussian marginal observations, and, importantly, includes measures of uncertainty and an accessible, publicly-available software package. We applied DPGP to existing data from a microbial model organism exposed to stress. We found that DPGP accurately recapitulated previous knowledge of TF-mediated gene regulation in response to H_2_O_2_ with minimal user input. We applied DPGP to a novel RNA-seq time series data set detailing the transcriptional response to dex in a human cell line. Our clusters identified four major response types: quickly up-regulated, slowly up-regulated, quickly down-regulated, and slowly down-regulated genes. These response types differed in TF binding and histone modifications before dex treatment and in changes in TF binding following dex treatment, indicating shared biological processes among genes in the same response cluster.

As with all statistical models, DPGP makes a number of assumptions about observations. In particular, DPGP assumes i) cluster trajectories are stationary; ii) cluster trajectories are exchangeable; iii) each gene belongs to only one cluster; iv) expression levels are sampled at the same time points across all genes; and v) the time point-specific residuals have a Gaussian distribution. Despite these assumptions, our results show that DPGP is robust to certain violations. In the human cell line data, exposure to dex resulted in a non-stationary response (at time point lag 1, all dex-responsive genes had either Augmented Dickey-Fuller *p* < 0.05 or Kwiatkowski—Phillips—Schmidt—Shin *p* > 0.05), and the residuals did not follow a Gaussian distribution (Schapiro-Wilk test, *p* ≤ 2.2 × 10^−16^), violating assumptions (i) and (v). However, despite these assumption violations, we found that DPGP clustered expression trajectories in a robust and biologically interpretable way. Furthermore, because DPGP does not assume that the gene expression levels are observed at identical intervals across time, DPGP allows study designs with non-uniform sampling.

Our DPGP model can be readily extended or interpreted in additional ways. For example, DPGP returns not only the cluster-specific mean trajectories but also the covariance of that mean, which is useful for downstream analysis by explicitly specifying confidence intervals around interpolated time points. Given the Bayesian framework, DPGP naturally allows for quantification of uncertainty in cluster membership by analysis of the posterior similarity matrix. For example, we could test for association of latent structure with specific genomic regulatory elements after integrating over uncertainty in the cluster assignments [39]. DPGP can also be applied to time series data from other types of sequencing-based genomics assays such as DNase-seq and ChIP-seq. If we find that the Gaussian assumption is not robust for alternative data types, we may consider using different distributions to model the response trajectories, such as a Student-*t* process [71].

When DPGP was applied to RNA-seq data from A549 cells exposed to GCs, the clustering results enabled several important biological observations. Two down-regulated response types were distinguished from one another based on histone marks and TF binding prior to GC exposure. The rapidly down-regulated cluster included homeobox TFs and growth factor genes and was enriched for basal enhancer regulatory activity. In contrast, slowly down-regulated cluster included critical cell cycle genes and was enriched for basal promoter regulatory activity. More study is needed to resolve how GCs differentially regulate these functionally distinct classes of genes. GR tends to bind distally from promoters [42] so that rapid down-regulation may be a direct effect of GR binding, while slower down-regulation may be secondary effect. We also found that down-regulated genes lost binding of transcriptional activators in distal regions, while up-regulated genes gained binding. This result links genomic binding to GC-mediated repression on a genome-wide scale. With increasing availability of high-throughput sequencing time series data, we anticipate that DPGP will be a powerful tool for characterizing cellular response types.

## Materials and methods

### Dirichlet process mixture model of Gaussian processes

We developed a Bayesian nonparametric model (S9 Fig) for time series trajectories $Y\in R^{P\times T}$, where *P* is the number of genes and *T* the number of time points per sample, assuming observations at the same time points across samples, but allowing for missing observations. In particular, let *y*_*j*_ be the vector of gene expression values for gene *j* ∈ {1, …, *P*} for all assayed time points *t* ∈ {1, …, *T*}.

Then, we define the generative DP mixture model as follows:

$$G∼DP(\alpha ,G_{0});$$ (1)

$$\theta_{h}∼G;$$ (2)

$$y_{j}∼p(\cdot |\theta_{h}).$$ (3)

Here, *DP* represents a draw *G* from a DP with *base distribution* *G*_0_. *G*, then, is the distribution from which the latent variables *θ*_*h*_ are generated for cluster *h*, with *α* > 0 representing the *concentration parameter*, with larger values of *α* encouraging more and smaller clusters. We specify the observation distribution *y*_*j*_ ∼ *p*(⋅|*θ*_*h*_) with a Gaussian process. With the DP mixture model, we are able to cluster the trajectory of each gene over time without specifying the number of clusters *a priori*.

Using exchangeability, we can integrate out *G* in the DP to find the conditional distribution of one cluster-specific random variable *θ*_*h*_ conditioned on all other variables *θ*_¬*h*_, which represent the cluster-specific parameter values of the observation distribution (here, a GP). This allows us to describe the distribution of each parameter conditioned on all others; for all clusters *h* ∈ {1, …, *H*} we have

$$p(\theta_{H}|\theta_{1},\ldots ,\theta_{H-1})\propto \alpha p\left(\theta_{H}|G_{0}\right)+\sum_{h=1}^{H-1}\delta_{\theta_{h}}\left(\theta_{H}\right),$$ (4)

where *δ*_*θ*_*h*__(⋅) is a Dirac delta function at the parameters for the *h*^*th*^ partition. A prior could be placed on *α*, and the posterior for *α* could be estimated conditioned on the observations. Here we favor simplicity and speed, and we set *α* to one. This choice has been used in gene expression clustering [19] and other applications [72, 73] and favors a relatively small number of clusters, where the expected number of clusters scales as *α*log*P*.

### Gaussian process prior distribution

Our base distribution for the DP mixture model captures the distribution of each parameter of the cluster-specific GP. A GP is a distribution on arbitrary functions mapping points in the input space *x*_*t*_—here, time—to a response *y*_*j*_—here, gene expression levels of gene *j* across time *t* ∈ {1, …, *T*}. The within-cluster parameters for the distribution of trajectories for cluster *h*, or $\theta_{h}=\left\{\mu_{h},\ell_{h},\tau_{h},\sigma_{h}^{2}\right\}$, can be written as follows:

$$\mu_{h}∼GP(\mu_{0},K)$$ (5)

$$\ell_{h}∼\ln N(0,1)$$ (6)

$$\tau_{h}∼\ln N(0,1)$$ (7)

$$\sigma_{h}^{2}∼\text{InverseGamma}(\alpha^{IG},\beta^{IG})$$ (8)

where *α*^*IG*^ captures shape and *β*^*IG*^ represents rate (inverse of scale). The above hyperparameters may be changed by the user of the DPGP software. By default, *α*^*IG*^ is set to 12 and *β*^*IG*^ is set to 2, as these were determined to work well in practice for our applications. For data with greater variability, such as microarray data, the shape parameter can be decreased to allow for greater marginal variance within a cluster. The base distributions of the cluster-specific parameters, which we estimate from the data, were chosen to be the conjugate prior distributions.

The positive definite Gram matrix *K* varies by cluster and quantifies the similarity between every pair of time points *x*, *x*′ in the absence of marginal variance using Mercer kernel function *K*_*h*,*t*,*t*′_ = *κ*_*h*_(*x*_*t*_, *x*_*t*′_). We used the squared exponential covariance function (dropping the gene index *j*):

$$\kappa_{h}\left(x_{t},x_{t^{\prime }}\right)=\tau_{h}^{2}\exp\{-\frac{\left|\right|x_{t}-x_{t^{\prime }}{\left|\right|}_{2}}{2\ell_{h}^{2}}\}.$$ (9)

The hyperparameter *ℓ*_*h*_, known as the *characteristic length scale*, corresponds to the distance in input space between which two data points have correlated outputs. The hyperparameter $\tau_{h}^{2}$, or *signal variance*, corresponds to the variance in gene expression trajectories over time. The model could be easily adapted to different choices of kernel functions depending on the stimulating conditions and the smoothness of the trajectories used in the analysis, such as the Matérn kernel [74], a periodic kernel [75], or a non-stationary kernel [76].

Including marginal (i.e., time point-specific) variance, $\sigma_{h}^{2}$ (Eq 8), the covariance between time points for trajectory *y*_*j*_ becomes $K_{h}+\sigma_{h}^{2}I$. Thus,

$$y_{j}∼N\left(\mu_{h},K_{h}+\sigma_{h}^{2}I\right),$$ (10)

where the marginal variance, $\sigma_{h}^{2}$, is unique to each cluster *h*. This specifies the probability distribution of each observation *y*_*j*_ in Eq (3) according to a cluster-specific GP.

### Markov chain Monte Carlo (MCMC) to estimate the posterior distribution of DPGP

Given this DPGP model formulation, we now develop methods to estimate the posterior distribution of the model parameters. We use MCMC methods, which have been used previously in time series gene expression analysis [19, 22]. MCMC allows the inference of cluster number and parameter estimation to proceed simultaneously. MCMC produces an estimate of the full posterior distribution of the parameters, allowing us to quantify uncertainty in their estimates. For MCMC, we calculate the probability of the trajectory for gene *j* belonging to cluster *h* according to the DP prior with the likelihood that gene *j* belongs to class *h* according to the cluster-specific GP distribution. We implemented Neal’s Gibbs Sampling “Algorithm 8” to estimate the posterior distribution of the trajectory class assignments [77]. More precisely, let *c*_*j*_ be a categorical latent variable specifying to what cluster gene *j* is assigned, and let *c*_¬*j*_ represent the class assignment vector for all trajectories except for gene *j*.

Using Bayes’ rule, we compute the distribution of each *c*_*j*_ conditioned on the data and all other cluster assignments:

$$Pr\left(c_{j}=h|y_{j},c_{\neg j},\theta_{h},\alpha \right)\propto Pr\left(c_{j}=h|c_{\neg j},\alpha \right)Pr\left(y_{j}|c_{j}=h,\theta_{h}\right)$$ (11)

where the first term on the right-hand side represents the probability of assigning the trajectory to cluster *h* and the second term represents the likelihood that the trajectory *y*_*j*_ was generated from the GP distribution for the *h*^*th*^ cluster.

According to our model specification, the probability *Pr*(*c*_*j*_ = *h*|*c*_¬*j*_, *α*) in Eq (11) is equivalent to the Chinese restaurant process in which:

$$\begin{array}{l} Pr(c_{j}=h|c_{\neg j},\alpha )\propto \\ \{\begin{array}{ll} \frac{\alpha /m}{\alpha +n-1} & \text{if}\;h\;\text{is}\;\text{empty}\;\text{or}\;\text{gene}\;j\;\text{assigned}\;\text{to}\;\text{singleton}\;\text{cluster}.\\ \frac{\sum_{j=1}^{n}1\left(c_{j}=h\right)}{\alpha +n-1} & \text{otherwise}. \end{array} \end{array}$$ (12)

In the above, *m* is the number of empty clusters available in each iteration. Similarly, the likelihood *Pr*(*y*_*j*_|*c*_*j*_ = *h*, *θ*_*h*_) in Eq (11) is calculated using our cluster-specific GPs:

$$\begin{array}{l} Pr(y_{j}|c_{j}=h,\theta_{h})=\\ \{\begin{array}{ll} N(y_{j}|\mu_{0}\left(x\right),K_{0}+\sigma_{0}^{2}I) & \text{if}\;h\;\text{is}\;\text{empty}\;\text{or}\;\text{gene}\;j\;\text{assigned}\;\text{to}\;\text{singleton}\;\text{cluster}.\\ N(y_{j}|\mu_{h}\left(x\right),K_{h}+\sigma_{h}^{2}I) & \text{otherwise}. \end{array} \end{array}$$ (13)

We draw *μ*_0_(*x*) as a sample from the prior covariance matrix, and we put prior distributions on parameters $\tau_{h}^{2}$, *ℓ*_*h*_, and $\sigma_{h}^{2}$ (Eqs 6–8) and estimate their posterior distributions explicitly.

In practice, the first 48% of the prespecified maximum number of MCMC iterations is split into two equally sized burn-in phases. At initialization, each gene is assigned to its own cluster, which is parameterized by its mean trajectory and a squared exponential kernel with unit signal variance and unit length-scale [after the mean time interval between sampling points has been scaled to one unit so that the length scale distribution remains reasonable (Eq 6)]. The local variance is initialized as the mode of the prior local variance distribution. During the first burn-in phase, a cluster is chosen for each gene at each iteration where the likelihood depends on the fit to a Gaussian process parameterized by the cluster’s mean function and the covariance kernel with initial parameters defined above.

Before each iteration, *m* empty clusters (by default, 4) are re-generated, each of which has a mean function drawn from the prior mean function *μ*_0_ with variance equivalent to the marginal variance described above. These empty clusters are also assigned the initial covariance kernel parameters described above.

After the second burn-in phase, we update the model parameters for each cluster at every *s*^*th*^ iteration to increase speed. Specifically, we compute the posterior probabilities of the kernel hyperparameters. To simplify calculations, we maximize the marginal likelihood, which summarizes model fit while integrating over the parameter priors, known as type II maximum likelihood [76]. The updated mean trajectory and covariance, respectively, then become:

$$\mu_{h}=K{\left(x,x\right)}_{h}{\left[K{\left(x,x\right)}_{h}+\sigma_{n,h}^{2}I\right]}^{-1}{\bar{y}}_{h}\;\text{where}\;{\bar{y}}_{h}=\frac{y_{1}+\cdots +y_{k}}{\sum_{j=1}^{n}1\left(c_{j}=h\right)},$$ (14)

$$K_{h}^{*}=K{\left(x^{\prime },x^{\prime }\right)}_{h}-K{\left(x^{\prime },x\right)}_{h}\left[K{\left(x,x\right)}_{h}+\sigma_{n,h}^{2}I\right]K{\left(x,x^{\prime }\right)}_{h},$$ (15)

for all expression trajectories {*y*_1_, …, *y*_*k*_} ∈ cluster *h*. We do this using the fast quasi-Newton limited-memory Broyden-Fletcher-Goldfarb-Shanno (L-BFGS) method implemented in SciPy [78]. After the second burn-in phase, the cluster assignment vector *c* is sampled at every *s*^*th*^ iteration to thin the Markov chain, where *s* = 3 by default. By default, the algorithm runs for 1,000 iterations. The algorithm can also check for convergence based on squared distance between the sampled partitions and the posterior similarity matrix and by change in posterior likelihood.

The version of statistical inference for DPGP is fully general in that it allows observations at different time points and missing data, which is a desirable feature of GP models. However, when the data are fully observed and the observations of the genes are made at identical time points, we can exploit the structure in the data for additional computational gains, as in related work [79]. In particular, we can use the marginal likelihood by gene to perform posterior inference instead of the marginal likelihood by cluster. This approach changes the model in that we now have separate estimates of the mean function for a cluster based on each gene, with those mean functions being drawn from a cluster-specific shared GP prior, as we make explicit in the generative model above. We refer to this version of inference for the DPGP as fDPGP. This marginalized approach reduces the complexity of the matrix inversion from $O({\left(MT\right)}^{3})$ for DPGP to $O(M^{3})$ for fDPGP for *M* genes and *T* time points. Note that in our application to the *H. salinarum* and dex exposure data we use fDPGP to scale to the data.

### Selecting the clusters

Our MCMC approach produces a sequence of states drawn from a Gibbs sampler, where each state captures a partition of genes into disjoint clusters. In DPGP, we allow several choices for summarizing results from the Markov chain. Here, we take the maximum *a posteriori* (MAP) clustering, or the partition that produces the maximum value of the posterior probability. We also summarize the information contained in the Gibbs samples into a *posterior similarity matrix* (PSM), *S*, of dimension *P* × *P*, for *P* genes, where *S*[*j*, *j*′] = the proportion of Gibbs samples for which a pair of genes *j*, *j*′ are in the same partition, i.e., $\frac{1}{Q}\sum_{q=1}^{Q}1\left[c_{j}^{q}=c_{j^{\prime }}^{q}\right]$, for *Q* samples and $c_{j}^{q}$ representing the cluster assignment of gene *j* in iteration *q*. This PSM avoids the problem of label switching by being agnostic to the cluster labels when checking for equality.

### Data simulations

In order to test our algorithm across a wide variety of possible data sets, we formulated more than twenty generative models with different numbers of clusters (10–100) and with different generative covariance parameters (signal variance 0.5–3, marginal variance 0.01–1, and length scale 0.5–3). We varied cluster number (data sets 1–5) and covariance parameters both across models and within models. For each model, we generated 20 data sets to ensure that results were robust to sampling. We simulated 620 data sets with Gaussian-distributed error and 500 data sets with *t*-distributed error for testing. To generate each data set, we specified the total number of clusters and the number of genes in each cluster. For each cluster, we drew the cluster’s mean expression from a multivariate normal (or multivariate *t*-distribution) with mean zero and covariance equivalent to a squared-exponential kernel with prespecified hyperparameter settings, then drew a number of samples (gene trajectories) from a multivariate normal (or multivariate *t*-distribution) with this expression trajectory as mean and the posterior covariance kernel as covariance.

We compared results of DPGP applied to these simulated data sets against results from six state-of-the-art methods, including two popular correlation-based methods and four model-based methods that use a finite GMM, infinite GMM, GPs, and spline functions.

- BHC (v.1.22.0) [25];
- GIMM (v.3.8) [21];
- hierarchical clustering by average linkage [11] [AgglomerativeClustering implemented in SciKitLearn (v.0.18.1) [80]];
- k-means clustering [13] [KMeans implemented in SciKitLearn (v.0.18.1) [80]];
- Mclust (v.4.4) [18];
- SplineCluster (v. Oct. 2010) [23].

Hierarchical clustering and k-means clustering were parameterized to return the true number of clusters. All of the above algorithms, including our own, were run with default arguments. The only exception was GIMM, which was run by specifying “complete linkage”, so that the number of clusters could be chosen automatically by cutting the returned hierarchical tree at distance 1.0, as in “Auto” IMM clustering [21].

We evaluated the accuracy of each approach using ARI. To compute ARI, let *a* equal the number of pairs of co-clustered elements that are in the same true class, *b* the number of pairs of elements in different clusters that are in different true classes, and *N* the total number of elements clustered:

$$\text{RI}=\frac{a+b}{\left(\begin{array}{c} N\\ 2 \end{array}\right)}$$ (16)

$$\text{ARI}=\frac{\text{RI}-E[\text{RI}]}{\max\left(\text{RI}\right)-E[\text{RI}]}$$ (17)

For a derivation of the expectation of RI above, see [32].

### Transcriptional response in *Halobacterium salinarum* control strain versus Δ*rosR* transcription factor knockout in response to H_2_O_2_

Gene expression microarray data from our previous study [30] (GEO accession GSE33980) were clustered using DPGP. In the experiment, *H. salinarum* control and Δ*rosR* TF deletion strains were grown under standard conditions (rich medium, 37°C, 225 r.p.m. shaking) until mid-logarithmic phase. Expression levels of all 2,400 genes in the *H. salinarum* genome [81] were measured in biological duplicate, each with 12 technical replicate measurements, immediately prior to addition of 25 mM H_2_O_2_ and at 10, 20, 40, 60, and 80 min after addition. Mean expression across replicates was standardized to zero mean and unit variance across all time points and strains. Standardized expression trajectories of 616 non-redundant genes previously identified as differentially expressed in response to H_2_O_2_ [30] were then clustered using DPGP with default arguments, except that the $\sigma_{n}^{2}$ hyperprior parameters were set to *α*^*IG*^ = 6 and *β*^*IG*^ = 2 to allow modeling of increased noise in microarray data relative to RNA-seq. Gene trajectories for each of the control and Δ*rosR* strains were clustered in independent DPGP modeling runs. Resultant clusters were analyzed to determine how each gene changed cluster membership in response to the *rosR* mutation. We computed the Pearson correlation coefficient in mean trajectory between all control clusters and all Δ*rosR* clusters. Clusters with the highest coefficients across conditions were considered equivalent across strains (e.g., control cluster 1 versus Δ*rosR* cluster 1, *ρ* = 0.886 in Fig 2). Significance of overrepresentation in cluster switching (e.g., from control cluster 1 to Δ*rosR* cluster 2) was tested using FET. To determine the degree of correspondence between DPGP results and previous clustering results with the same data, we took the intersection of the list of 372 genes that changed cluster membership according to DPGP with genes in each of eight clusters previously detected using k-means [30]. Significance of overlap between gene lists was calculated using FET.

### GC transcriptional response in a human cell line

A549 cells were cultured and exposed to the GC dex or a paired vehicle ethanol (EtOH) control as in previous work [42] with triplicates for each treatment and time point. Total RNA was harvested using the Qiagen RNeasy miniprep kit, including on column DNase steps, according to the manufacturer’s protocol. RNA quality was evaluated using the Agilent Tape station and all samples had a RNA integrity number > 9. Stranded Poly-A+ RNA-seq libraries were generated on an Apollo 324 liquid handling platform using the Wafergen poly-A RNA purification and RNA-seq kits according to manufacturer instructions. The resulting libraries were then pooled in equimolar ratios and sequenced on two lanes 50 bp single-end lanes on an Illumina HiSeq 2000. Data are available at GEO under study accession GSE104714.

RNA-seq reads were mapped to GENCODE (v.19) transcripts using Bowtie (v.0.12.9) [82] and quantified using samtools idxstats utility (v.1.3.1) [83]. Differentially expressed (DE) transcripts were identified in each time point separately using DESeq2 (v.1.6.3) [84] with default arguments and FDR ≤10%. We clustered only one transcript per gene, in particular, the transcript with the greatest differential expression over the time course among all transcripts for a given gene model, using Fisher’s method of combined p-values across time points. Further, we only clustered transcripts that were differentially expressed for at least two consecutive time points, similar to the approach of previous studies [7, 85]. We standardized all gene expression trajectories to have zero mean and unit variance across time points. We clustered transcripts with DPGP with default arguments.

To query the function of our gene expression clusters, we annotated all transcripts tested for differential expression with their associated biological process Gene Ontology slim (GO-slim) [51] terms and performed functional enrichment analysis using FET with FDR correction [86] as implemented in GOAtools [87]. We considered results significant with FDR ≤ 5%.

We compared DPGP clusters in terms of TF binding and histone modification occupancy as assayed by ChIP-seq (S5 Table). For each of the ENCODE BAM files whose root names are listed in S5 Table, we tallied read counts in flanking regions of the transcription start site (TSS) of the gene from which each transcript derived. Flanking regions were split into the following bins: within 1 kb of the TSS, 1–5 kb from the TSS, and 5–20 kb from the TSS; reads were quantified in those bins using the software featureCounts (v.1.4.6) [88]. For data sets in which two replicates were available, we merged mapped reads across replicates. We normalized counts by the total number of mapped reads. TF binding tends to be correlated (in enhancers and promoters, for example), as does histone modification occupancy. In order to determine the features relevant to the prediction of cluster membership, we chose to apply elastic net logistic regression, which combines lasso (*ℓ*_1_) and ridge (*ℓ*_2_) penalties. Elastic net tends to shrink to zero the coefficients of groups of correlated predictors that have little predictive power [59]. We ran regression models to predict cluster membership from log_10_ normalized counts of TF binding and histone modifications in control conditions (2% EtOH by volume and untreated) and, separately, from log_10_ fold-change in normalized TF binding from 2% EtOH by volume to 100 nM dex conditions. We used stochastic gradient descent as implemented in SciKitLearn [80] to efficiently estimate the parameters of our model. We searched for optimal values for the *ℓ*_1_/*ℓ*_2_ ratio and the regularization multiplier (*λ*) by fitting our model with 5-fold stratified cross-validation across a grid of possible values for both variables (*ℓ*_1_/*ℓ*_2_ ∈ {0.5, 0.75, 0.9, 0.95, 1}, *λ* ∈ {10^−6^, 10^−5^, 10^−4^, 10^−3^, 10^−2^, 1}). We selected the sparsest model (least number of non-zero coefficients) with mean log-loss within one standard error of the mean log loss of the best performing model [89].

We performed principal components analysis (as implemented in SciKitLearn [80]) on the standardized log_10_ library size-normalized binned counts of TF binding and histone modifications in control conditions only for the observations that corresponded to transcripts in the four largest DPGP clusters.

## Supporting information

S1 Fig Clustering performance of state-of-the-art algorithms on simulated time series data with *t*-distributed error. (A–H) Box plots show summaries of the empirical distribution of clustering performance for each method in terms of Adjusted Rand Index (ARI) across twenty instances of 25 data set types detailed in S1 Table, but with *t*-distributed error (*df* = 2). Vertical dotted lines separate data sets generated with widely varied cluster size distributions (*left*) from data sets generated with widely varied generating hyperparameters (*right*). Observations that lie beyond the first or third quartile by 1.5× the interquartile range are shown as outliers.
(TIF)

S2 Fig Clustering performance after excluding genes by estimated minimum probability of inclusion in assigned cluster. Box plots show distribution of ARI across varied gene-to-cluster inclusion probabilities for (A) all data sets in S1 Table; (B) after permuting probabilities of inclusion; for (C) selected data sets in S1 Table (4, 5, 13–17, 29–31); and (D) after permuting probabilities. (E) Box plots show distribution of difference in ARI computed for all clustered genes and ARI computed only for genes with probability of cluster inclusion > 0.9 for all data sets in S1 Table.
(TIF)

S3 Fig Time benchmark. (A) Mean runtime of BHC, GIMM, DPGP, and fDPGP across varying numbers of gene expression trajectories generated from GPs parameterized in the same manner as simulated data sets 11, 21, and 27 in S1 Table. There were 2, 4, 8, 16, 32, and 64 simulated genes per cluster and there were 1–8 different clusters per cluster size. Error bars represent standard deviation in runtime across 20 simulated data sets. Hierarchical clustering, k-means, Mclust, and SplineCluster are not shown because their mean runtimes were under one minute and could not be meaningfully displayed here. (B) Same as (A) but with 10 simulated genes per cluster for 10 clusters and an additional 100 simulated genes per cluster for the remainder of the total number of simulated genes. Standard deviation in runtime computed across 10 simulated data sets.
(TIF)

S4 Fig Proportion of held-out test points within credible intervals of estimated cluster means for DPGP. For all data sets detailed in S1 Table, expression trajectories were clustered while separately holding out each of the four middle time points of eight total time points. Box plot shows proportion of test points that fell within the 95% credible intervals (CIs) of the estimated cluster mean.
(TIF)

S5 Fig Rugplot of all cluster sizes for A549 glucocorticoid exposure data clustered using DPGP. Each stick on the x-axis represents a singular data cluster of the 13 total clusters. Note that the two clusters with sizes 22 and 23 are difficult to distinguish by eye.
(TIF)

S6 Fig Non-zero regression coefficients in the prediction of cluster membership for four largest DPGP clusters. Heatmap shows all coefficients (sorted by sum of absolute value across clusters) estimated by elastic net logistic regression of cluster membership for the four largest DPGP clusters as predicted by log_10_ normalized binned counts of ChIP-seq TF binding and histone modifications in control conditions. Distance indicated in row names reflects the bin of the predictor (e.g. < 1 kb = within 1 kb of TSS).
(TIF)

S7 Fig Non-zero regression coefficients in the prediction of cluster membership for down-regulated DPGP clusters. All non-zero coefficients estimated by elastic net logistic regression of cluster membership for two largest down-regulated DPGP clusters on TF binding and histone modifications in A549 cells in control conditions. Distance indicated in row names reflects the bin of the predictor (e.g., 1 kb = within 1 kb of TSS).
(TIF)

S8 Fig Scree plot of percentage of variance explained by each principal component in decomposition of ChIP-seq matrix. The log_10_ normalized ChIP-seq binned counts around the TSS of genes representing TF binding and histone modification occupancy in control conditions was decomposed by PCA. The percentage of variance explained by each of the top ten PCs is shown here.
(TIF)

S9 Fig Plate model of DPGP sampling method. Variables are as described in Materials and methods.
(TIF)

S1 Table Simulated data sets used for algorithm comparisons. (XLSX)

S2 Table P-values for algorithm comparisons on simulated data. (XLSX)

S3 Table Frequency of switches observed in DPGP clusterings from control to Δ*rosR* deletion mutant in H_2_O_2_ exposure in ***H. salinarum***. (XLSX)

S4 Table Functional enrichment results for four largest DPGP expression clusters in A549 cells in response to the glucocorticoid dexamethasone. (XLSX)

S5 Table ENCODE ChIP-seq data sets used in the analysis of GC-responsive clusters. (XLSX)

S6 Table Principal components analysis loadings by feature for PC1 and PC2 for ChIP-seq TF binding and histone modifications in A549 cells in control conditions. (XLSX)
