# Supplementary figures and images for: Clustering gene expression time series data using an infinite Gaussian process mixture model
Source: PLoS Comput Biol. 2018 Jan 16;14(1):e1005896. doi: 10.1371/journal.pcbi.1005896 (PMC5786324; doi:10.1371/journal.pcbi.1005896)

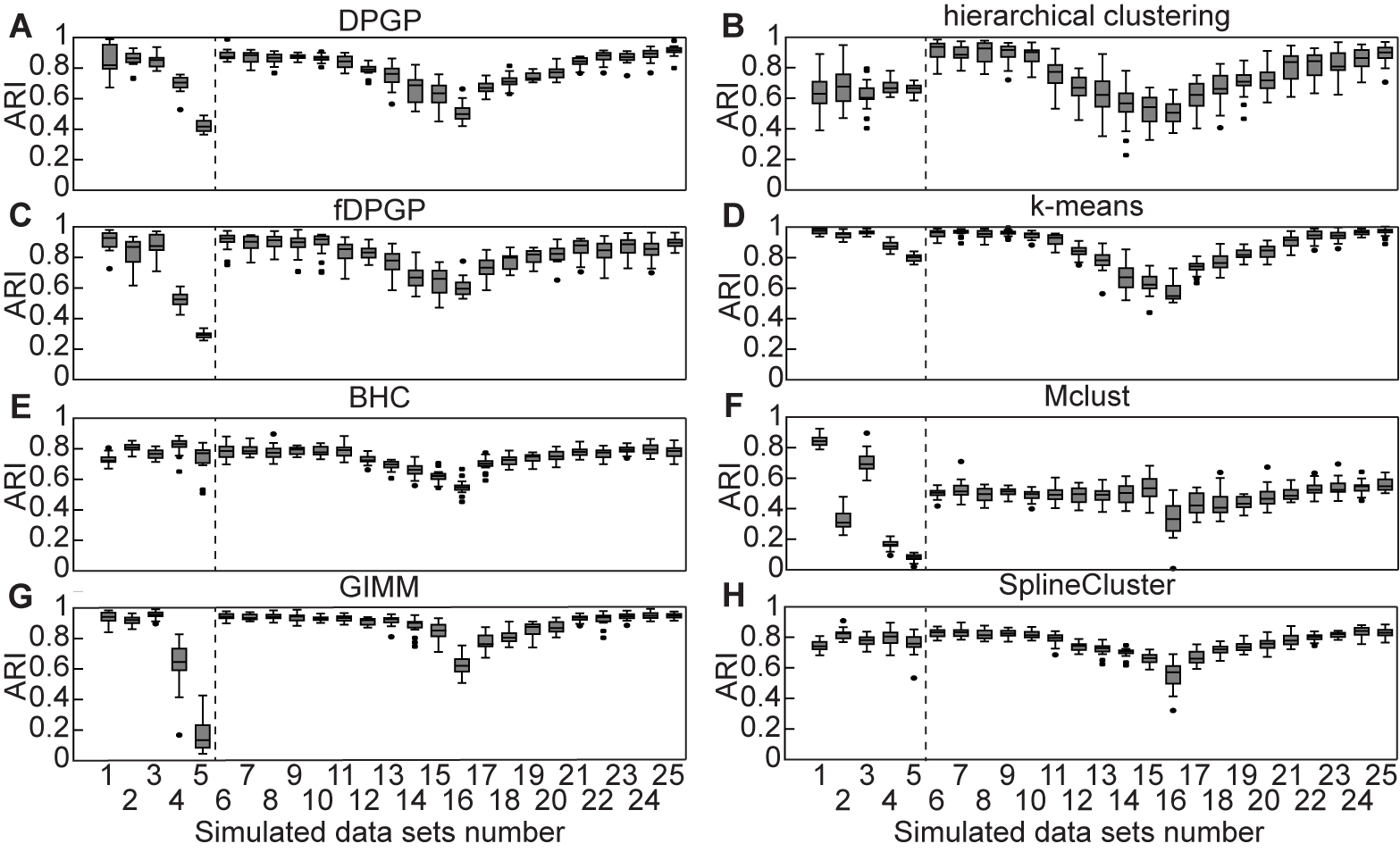

Supplement: S1 Fig — (A–H) Box plots show summaries of the empirical distribution of clustering performance for each method in terms of Adjusted Rand Index (ARI) across twenty instances of 25 data set types detailed in S1 Table, but with t-distributed error (df = 2). Vertical dotted lines separate data sets generated with widely varied cluster size distributions (left) from data sets generated with widely varied generating hyperparameters (right). Observations that lie beyond the first or third quartile by 1.5× the interquartile range are shown as outliers. (TIF) [file pcbi.1005896.s001.tif]

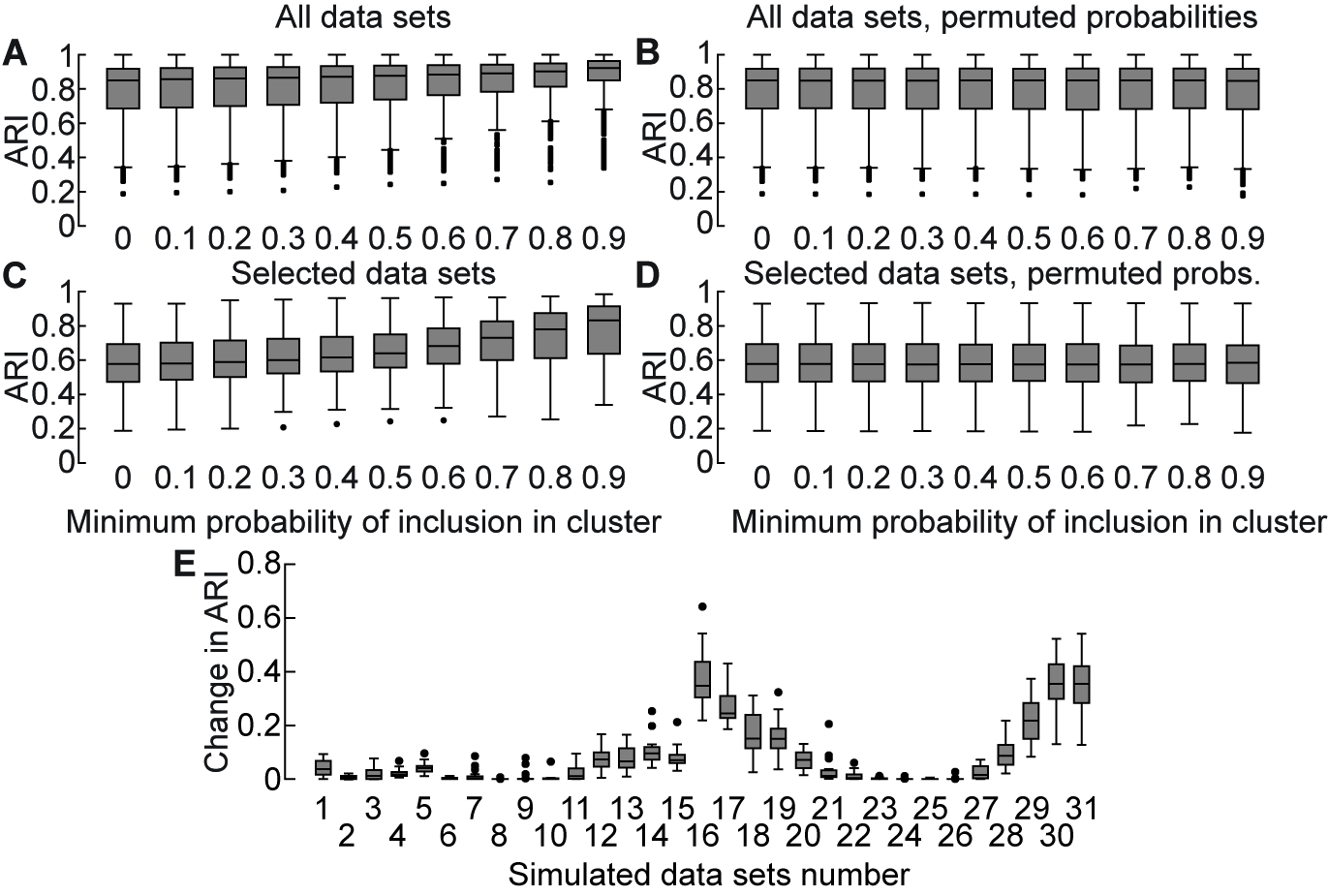

Supplement: S2 Fig — Box plots show distribution of ARI across varied gene-to-cluster inclusion probabilities for (A) all data sets in S1 Table; (B) after permuting probabilities of inclusion; for (C) selected data sets in S1 Table (4, 5, 13–17, 29–31); and (D) after permuting probabilities. (E) Box plots show distribution of difference in ARI computed for all clustered genes and ARI computed only for genes with probability of cluster inclusion > 0.9 for all data sets in S1 Table. (TIF) [file pcbi.1005896.s002.tif]

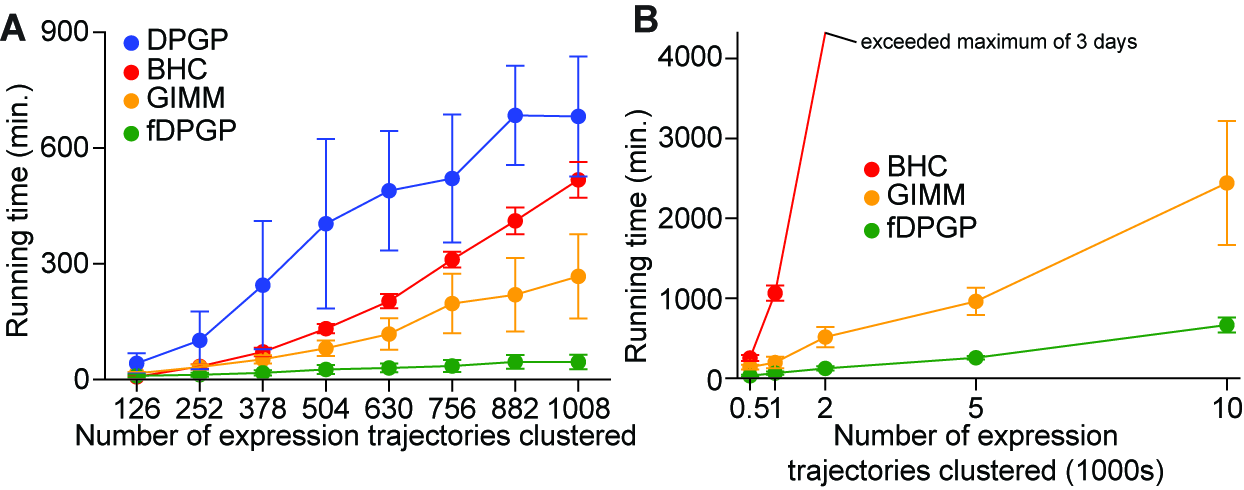

Supplement: S3 Fig — (A) Mean runtime of BHC, GIMM, DPGP, and fDPGP across varying numbers of gene expression trajectories generated from GPs parameterized in the same manner as simulated data sets 11, 21, and 27 in S1 Table. There were 2, 4, 8, 16, 32, and 64 simulated genes per cluster and there were 1–8 different clusters per cluster size. Error bars represent standard deviation in runtime across 20 simulated data sets. Hierarchical clustering, k-means, Mclust, and SplineCluster are not shown because their mean runtimes were under one minute and could not be meaningfully displayed here. (B) Same as (A) but with 10 simulated genes per cluster for 10 clusters and an additional 100 simulated genes per cluster for the remainder of the total number of simulated genes. Standard deviation in runtime computed across 10 simulated data sets. (TIF) [file pcbi.1005896.s003.tif]

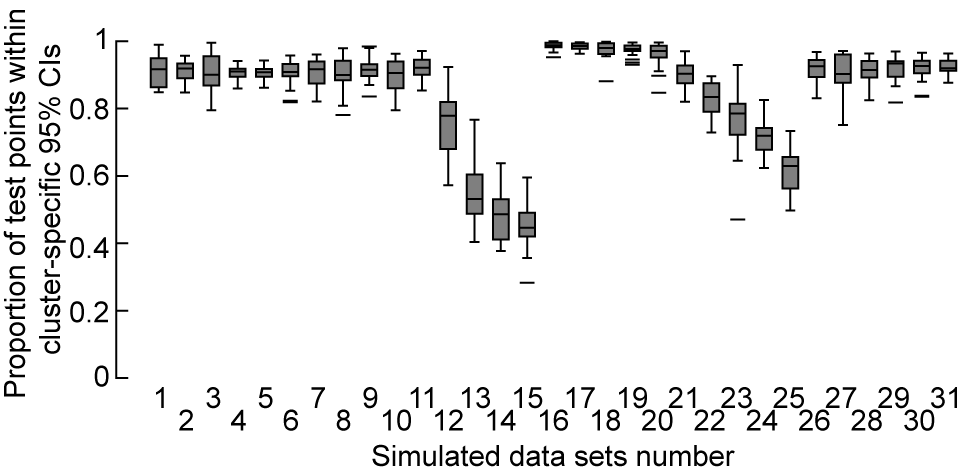

Supplement: S4 Fig — For all data sets detailed in S1 Table, expression trajectories were clustered while separately holding out each of the four middle time points of eight total time points. Box plot shows proportion of test points that fell within the 95% credible intervals (CIs) of the estimated cluster mean. (TIF) [file pcbi.1005896.s004.tif]

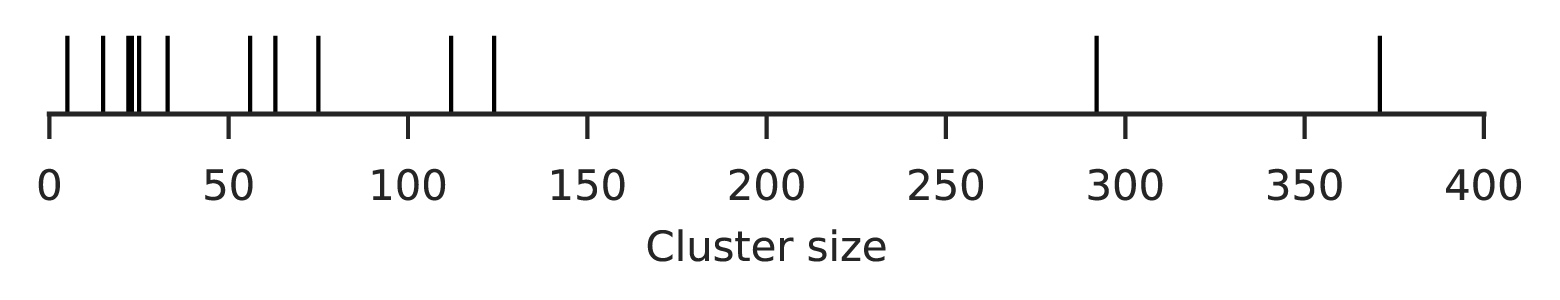

Supplement: S5 Fig — Each stick on the x-axis represents a singular data cluster of the 13 total clusters. Note that the two clusters with sizes 22 and 23 are difficult to distinguish by eye. (TIF) [file pcbi.1005896.s005.tif]

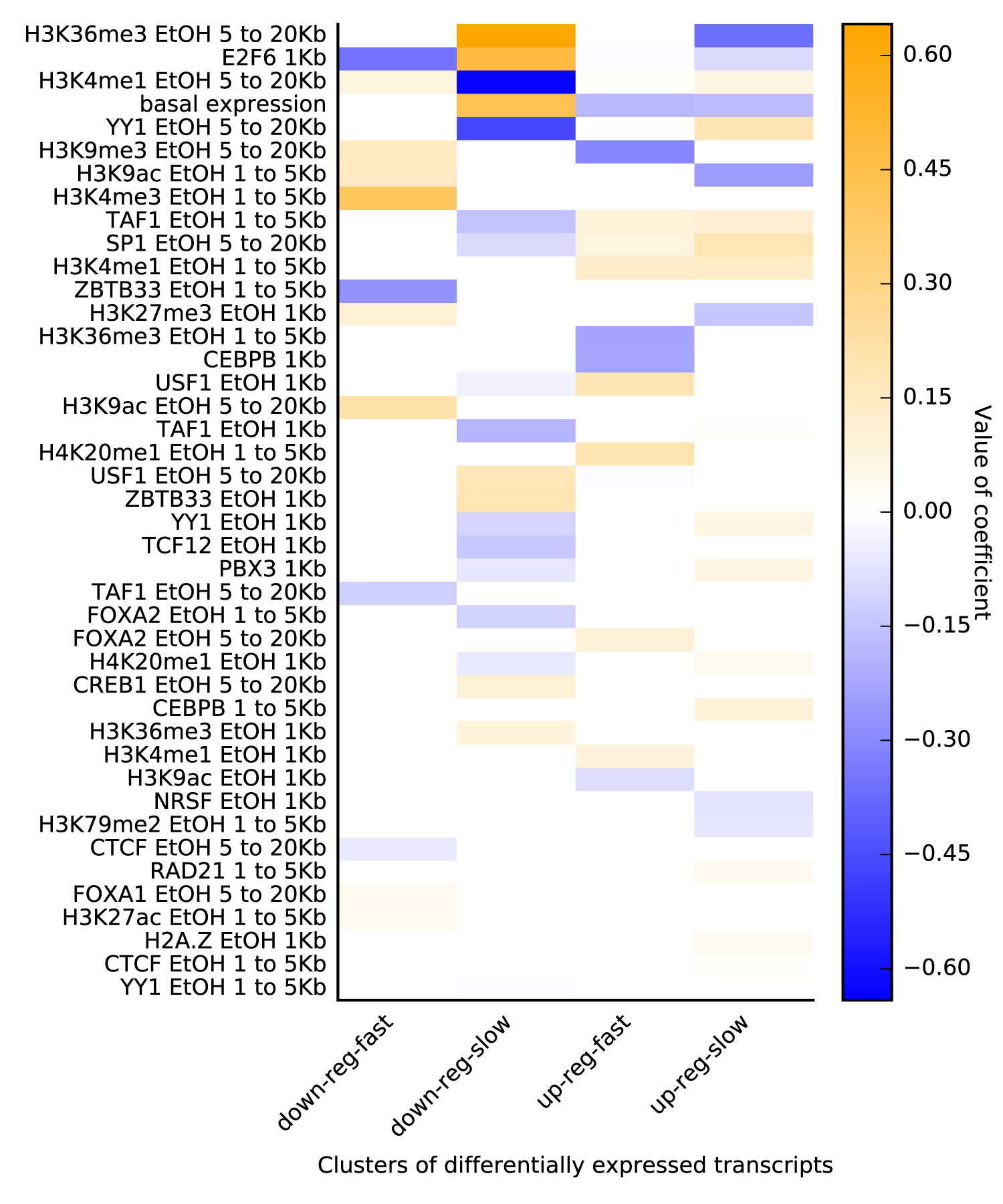

Supplement: S6 Fig — Heatmap shows all coefficients (sorted by sum of absolute value across clusters) estimated by elastic net logistic regression of cluster membership for the four largest DPGP clusters as predicted by log10 normalized binned counts of ChIP-seq TF binding and histone modifications in control conditions. Distance indicated in row names reflects the bin of the predictor (e.g. < 1 kb = within 1 kb of TSS). (TIF) [file pcbi.1005896.s006.tif]

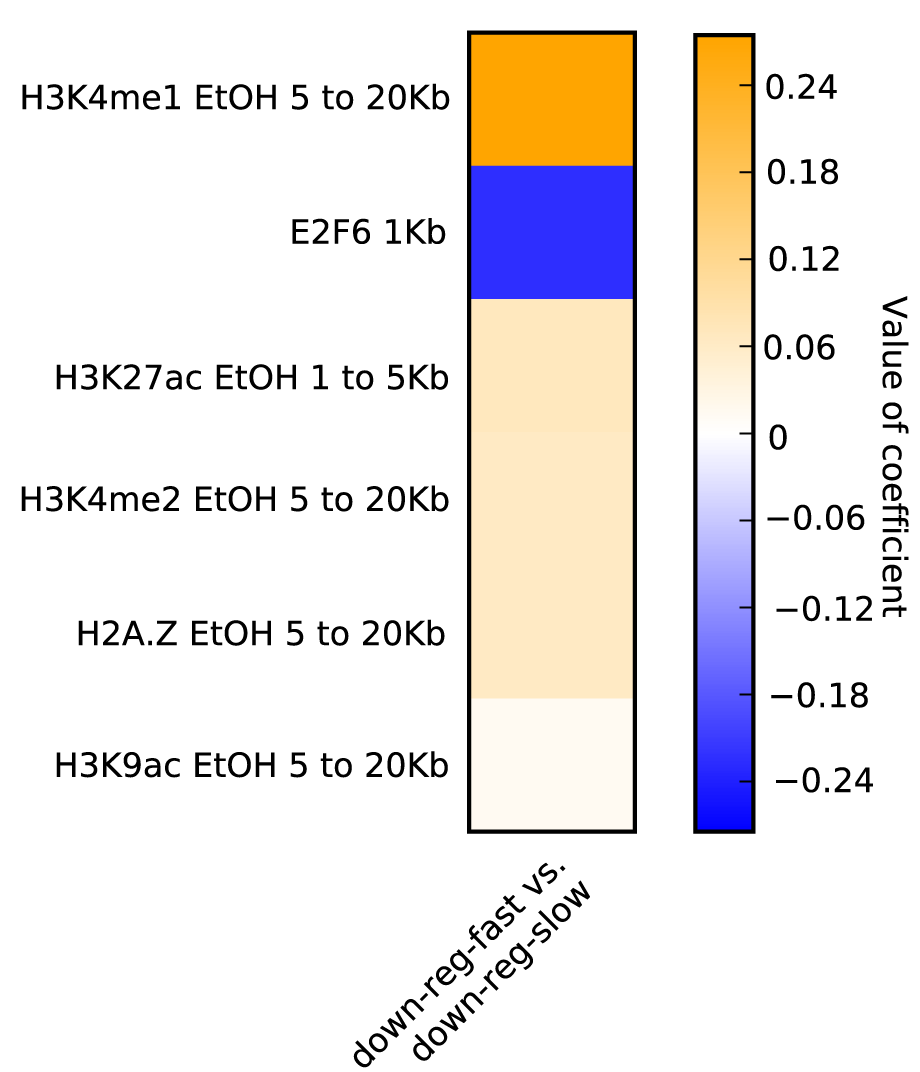

Supplement: S7 Fig — All non-zero coefficients estimated by elastic net logistic regression of cluster membership for two largest down-regulated DPGP clusters on TF binding and histone modifications in A549 cells in control conditions. Distance indicated in row names reflects the bin of the predictor (e.g., 1 kb = within 1 kb of TSS). (TIF) [file pcbi.1005896.s007.tif]

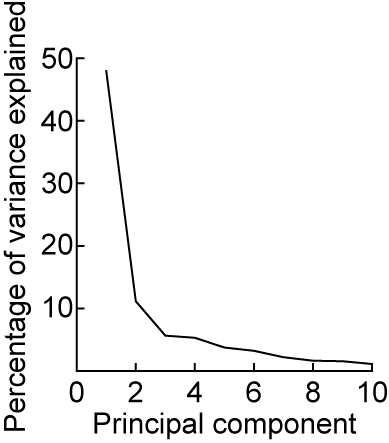

Supplement: S8 Fig — The log10 normalized ChIP-seq binned counts around the TSS of genes representing TF binding and histone modification occupancy in control conditions was decomposed by PCA. The percentage of variance explained by each of the top ten PCs is shown here. (TIF) [file pcbi.1005896.s008.tif]

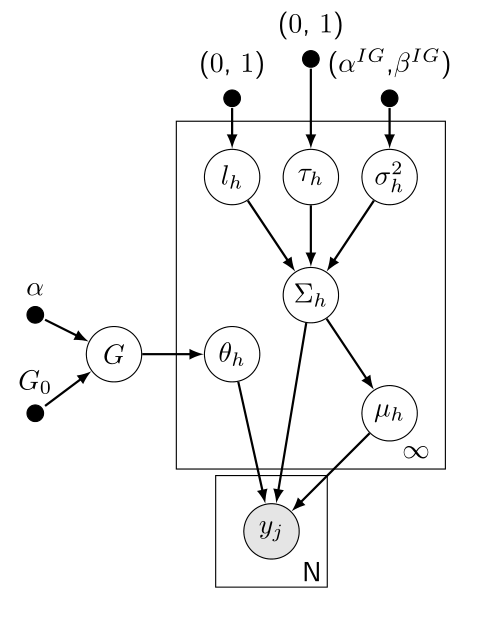

Supplement: S9 Fig — Variables are as described in Materials and methods. (TIF) [file pcbi.1005896.s009.tif]
